# Physiology and Pharmacology of Effects of GLP-1-based Therapies on Gastric, Biliary and Intestinal Motility

**DOI:** 10.1210/endocr/bqae155

**Published:** 2024-11-21

**Authors:** Ryan J Jalleh, Chinmay S Marathe, Christopher K Rayner, Karen L Jones, Mahesh M Umapathysivam, Tongzhi Wu, Daniel R Quast, Mark P Plummer, Michael A Nauck, Michael Horowitz

**Affiliations:** Endocrine and Metabolic Unit, Royal Adelaide Hospital, Adelaide, SA 5000, Australia; Adelaide Medical School, The University of Adelaide, Adelaide, SA 5000, Australia; Endocrine and Metabolic Unit, Royal Adelaide Hospital, Adelaide, SA 5000, Australia; Adelaide Medical School, The University of Adelaide, Adelaide, SA 5000, Australia; Adelaide Medical School, The University of Adelaide, Adelaide, SA 5000, Australia; Department of Gastroenterology and Hepatology, Royal Adelaide Hospital, Adelaide, SA 5000, Australia; Endocrine and Metabolic Unit, Royal Adelaide Hospital, Adelaide, SA 5000, Australia; Adelaide Medical School, The University of Adelaide, Adelaide, SA 5000, Australia; Endocrine and Metabolic Unit, Royal Adelaide Hospital, Adelaide, SA 5000, Australia; Adelaide Medical School, The University of Adelaide, Adelaide, SA 5000, Australia; Southern Adelaide Diabetes and Endocrine Service, Flinders Medical Centre, Bedford Park, SA 5042, Australia; Adelaide Medical School, The University of Adelaide, Adelaide, SA 5000, Australia; Diabetes, Endocrinology, Metabolism Section, Medical Department I, Katholisches Klinikum Bochum gGmbH, Sankt Josef-Hospital, Ruhr-University, D-44791 Bochum, Germany; Adelaide Medical School, The University of Adelaide, Adelaide, SA 5000, Australia; Intensive Care Unit, Royal Adelaide Hospital, Adelaide, SA 5000, Australia; Diabetes, Endocrinology, Metabolism Section, Medical Department I, Katholisches Klinikum Bochum gGmbH, Sankt Josef-Hospital, Ruhr-University, D-44791 Bochum, Germany; Institute for Clinical Chemistry and Laboratory Medicine, University Medicine Greifswald, D-17475 Greifswald, Germany; Endocrine and Metabolic Unit, Royal Adelaide Hospital, Adelaide, SA 5000, Australia; Adelaide Medical School, The University of Adelaide, Adelaide, SA 5000, Australia

**Keywords:** biliary, gastric emptying, glucagon-like peptide-1, hypoglycemia, physiology, small intestinal motility

## Abstract

Glucagon-like peptide-1 (GLP-1) receptor agonists and the dual GLP-1- and glucose-dependent insulinotropic polypeptide receptor co-agonist tirzepatide (referred to here collectively as “GLP-1-based therapy”) are incretin-based therapies being used increasingly in the management of both type 2 diabetes and obesity. They are now recognized to have beneficial effects beyond improved glycemic control and weight loss, including cardiovascular and renal protection. GLP-1-based therapy also slows gastric emptying, which has benefits (lowering postprandial glucose), but also potential risks (eg, hypoglycemia in individuals on insulin or sulphonylurea therapy). Their effects on the gallbladder may also be beneficial, contributing to reducing postprandial triglycerides, but they also potentially increase the risk of biliary disease. In this review, we summarize the effects of GLP-1 and incretin-based therapeutics on gastric, biliary and small intestinal function. An improved understanding of these effects will optimize the use of these drugs.

The use of glucagon-like peptide-1 (GLP-1)-based therapies, including GLP-1 receptor agonists (RAs) and tirzepatide, is increasing, based on their benefits for cardiovascular and renal health, in addition to their well-established capacity to improve glycemic control in individuals with type 2 diabetes (T2D) (1, 2) and induce weight loss (3). More recently, clinical trials involving drugs that combine GLP-1 receptor agonism with agonism or antagonism of other receptors, such as those for GIP or glucagon, have shown considerable promise in the management of T2D (4) and obesity (5) (eg, the triple GLP-1/GIP/glucagon RA, retatrutide). Small molecule oral GLP-1RAs in development, like orforglipron, also provide for easier administration (6). An understanding of the effects of GLP-1-based therapy on gastrointestinal function, which are integral to many of their benefits, as well as their adverse effects, is important. Potential harms associated with GLP-1-based therapies to slow gastric emptying have recently received considerable attention, including in mainstream media (7). A recent review by Drucker (8) provides a broad-based review on the efficacy and safety of GLP-1RAs, including potential novel therapeutic applications. Our review is focused on current knowledge of the effects of GLP-1-based therapies on gastric emptying, biliary/intestinal motility, and the implications for their clinical use.

## Physiology of Gastric Emptying

Gastroduodenal motility is modulated by the interstitial cells of Cajal and fibroblast-like cells that stain positively for platelet-derived growth factor receptor alpha (9). These pacemaker cells are electrically coupled to the smooth muscle cells of the stomach by gap junctions to regulate motor activity (9). Patterns of contractile activity in the proximal stomach, antrum, and pylorus differ between the interdigestive (fasting) and the postprandial states (10). The interdigestive state, characterized by cyclical gastric and small intestinal motility—the “migrating motor complex”—has 4 phases (11). During phase I, there is motor quiescence lasting for ∼40 minutes and this is followed by phase II, associated with intermittent contractions of ∼50 minutes’ duration (10). Phase III, which lasts 5 to 10 minutes, is characterized by large amplitude regular contractions, during which larger nondigestible solids (including tablets/capsules that are not broken down) are expelled from the stomach and the upper small intestine (10). Phase IV is a short quiescent transition period (11). The migrating motor complex is converted to a postprandial pattern by the arrival of food in the stomach. The latter is characterized by a reduction in proximal gastric tone to accommodate the meal, whereas antral contractions grind digestible solid contents against a closed pylorus until they are reduced to particles 1 to 2 mm in size before emptying from the stomach (10).

In health, digestible solids and nutrient-containing liquids empty more slowly than nonnutrient liquids. There is a large interindividual (but much lesser intraindividual) variation in gastric emptying of nutrients, with the rate dependent on the caloric content of ingested food, ranging from 1 to 4 kcal per minute (12). This variability is even greater in obesity and both type 1 diabetes (T1D) and T2D (13). In obese individuals without T2D, and those with recently diagnosed with well-controlled T2D, gastric emptying is usually either normal or modestly accelerated (14-16), whereas in longstanding and complicated T2D and T1D, it is delayed in ∼30% of individuals (13). There are also sex and ethnic differences in the rate of gastric emptying with emptying being slower in women with T2D compared to men (17) and in Whites compared to Han Chinese (18).

## The Impact of Gastric Emptying on Postprandial Glycemia and Energy Intake

**Figure 1.**
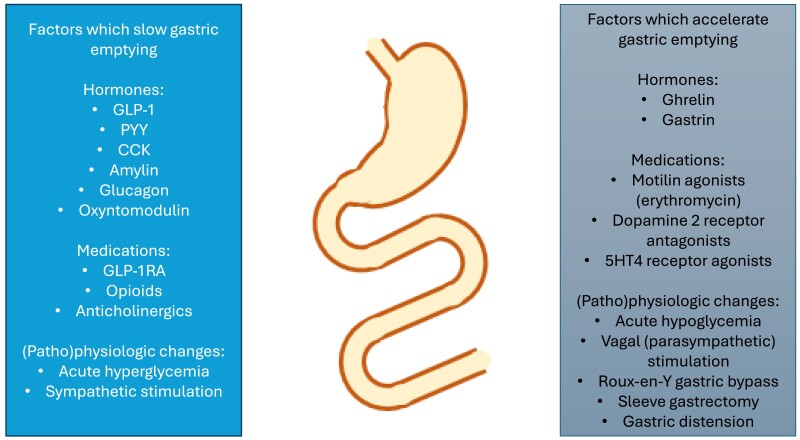
Factors that slow or accelerate gastric emptying.

The relationships between gastric emptying, postprandial glycemic excursions, and energy intake are complex and impacted by glucose tolerance status. In individuals with normal glucose tolerance, the rise in plasma glucose during a 75-g oral glucose tolerance test at 30 minutes, but not 60 minutes, is related directly to the rate of gastric emptying, so that the rise is greater when gastric emptying is relatively more rapid. In contrast, at 120 minutes, the relationship is inverse (ie, the blood glucose level is lower when gastric emptying is faster), probably reflecting the antecedent higher levels of insulin that subsequently lower blood glucose (19). In contrast, in individuals with impaired glucose tolerance but not diabetes, the rise in plasma glucose at both 30 and 60 minutes, but not 120 minutes, is related directly to the rate of gastric emptying, whereas in individuals with T2D, plasma glucose levels at 30, 60, and 120 minutes are all related directly to the rate of gastric emptying (19, 20). This progressive “shift to the right” is likely to reflect the concomitant progressive impairment in insulin secretion and sensitivity. Relationships between the rise in plasma glucose following nutrients and gastric emptying have also been shown with carbohydrate-containing meals. For example, in individuals with T2D, the incremental rises in glucose (measured by a continuous glucose sensor) between 0 to 30 minutes, 0 to 60 minutes, and 0 to 120 minutes following carbohydrate-containing meals is greater when gastric emptying is more rapid (21). Accordingly, gastric emptying is unequivocally a major determinant of the postprandial glucose excursion in both health and diabetes, accounting for ∼35% of the variance in the initial rise (22). Relatively more rapid gastric emptying, by definition, leads to the delivery of nutrients to the small intestine at a greater velocity, where they stimulate the secretion of hormones that may suppress appetite, including GLP-1, peptide YY (PYY), cholecystokinin (CCK), and oxyntomodulin (23) (Fig. 1). Both GLP-1 and PYY, which are co-secreted from intestinal L cells (predominantly located in the distal small intestine), slow gastric emptying, constituting a negative feedback mechanism called the “ileal brake” (24). It should be appreciated that gastric emptying is slowed by acute elevations in blood glucose induced by hyperglycemic clamps (25), although there is evidence that the effect of spontaneous elevations in glucose may be less (15, 26). This is an issue which requires further clarification, particularly given its potential relevance to evaluation of the effect of GLP-1RAs on gastric emptying in diabetes.

Satiety and satiation are both important determinants of energy intake and related to gastric emptying. “Satiation” refers to the sensation of fullness during a meal that brings a period of eating to an end (27), whereas “satiety” refers to the period following a meal when hunger and further eating are inhibited (27). In healthy individuals, slower gastric emptying is associated with reduced energy intake at an *ad libitum* buffet meal (ie, increased satiation) (28). This relationship may reflect the effect of more sustained stimulation of mechanoreceptors in the stomach by gastric distension, to suppress appetite-regulating centers in the brain via vagal afferents (29). The intragastric distribution of food is an important determinant of energy intake, in that distension of the gastric antrum is associated with the sensation of fullness and predictive of subsequent energy intake at a meal (30), although distension of the proximal stomach may also be important (31). In summary, the rate of gastric emptying is directly associated with postprandial glycemia and inversely with energy intake.

## Methods for Measuring Gastric Emptying

**Figure 2.**
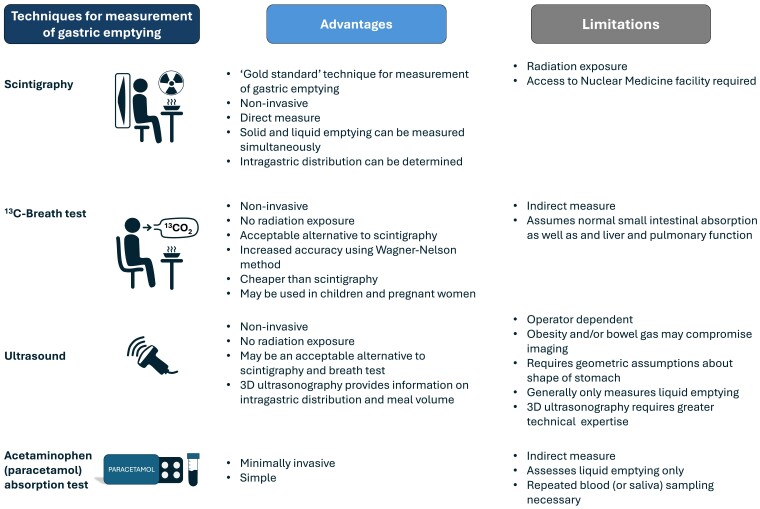
Techniques frequently used to measure gastric emptying.

When evaluating the effects of GLP-1RAs (or any other drug) on gastric emptying, accurate measurement is essential. Several techniques can measure gastric emptying precisely, but the majority of studies relating to GLP-1RAs have, unfortunately, employed suboptimal methodology. A summary of techniques frequently used to measure gastric emptying is presented (Fig. 2). Scintigraphy, a noninvasive technique (32), in which a gamma camera is used to measure emptying of radiolabeled food, remains the “gold standard” (13) and has a number of advantages. It provides a direct measure of gastric emptying and, by using 2 isotopes, allows for concurrent measurement of emptying of both solids and liquids (33). By drawing “regions of interest” to identify the proximal and distal stomach, scintigraphy also allows assessment of intragastric meal distribution (34, 35). Inherent limitations include the high cost, limited availability, and exposure to ionizing radiation. For example, the latter precludes studies during pregnancy.

The stable isotope breath test (36) is increasingly used in both clinical practice and research. Unlike scintigraphy, the test is not associated with a radiation burden. It is also noninvasive, simple, and readily available. Validation against the “gold standard” of scintigraphy has demonstrated strong correlations for measurements of solid and liquid emptying between the 2 techniques in both health (37, 38) and diabetes (39). With this method, a test meal is labeled with a ^13^C-substrate that is rapidly absorbed in the duodenum after leaving the stomach, oxidized in the liver and exhaled as ^13^CO_2_, which is collected via serial breath samples and measured using mass spectrometry. Gastric emptying is the rate-limiting step in the appearance of ^13^CO_2_ in the breath, and is estimated by the percentage of ^13^CO_2_ collected over time, utilizing a mathematical model. The original analytical models provided notional measurements of gastric half-emptying time (36), but more recent use of the Wagner-Nelson method, a pharmacokinetic model employing an elimination constant, provides precise measurements, comparable to scintigraphy (39). Limitations of the breath test include the assumption that the times required for small intestinal absorption and hepatic metabolism of isotope are negligible (ie, effects of gastric emptying and small intestinal motility cannot be discriminated), and that it is an indirect measure of gastric emptying (40).

Two-dimensional and 3D ultrasound techniques have been validated against scintigraphy (41-43). Ultrasound offers several advantages: it is noninvasive, not associated with a radiation burden, easy to perform with experience, usually provides images with high temporal and spatial resolution, and is readily available. With 3D ultrasound, intragastric meal distribution can also be evaluated. Limitations include the requirement for an experienced operator, the indirect inference of gastric emptying from changes in antral area or intragastric volume, and the challenge of imaging individuals with obesity or extensive intragastric gas. Neither the 2-dimensional or 3D technique is able to measure emptying of solids and liquids concurrently.

The acetaminophen (paracetamol) technique continues to be used frequently because of its simplicity and low cost. However, this technique has major limitations and we believe it should be avoided, particularly in isolation (44, 45). Acetaminophen is added to a test meal (usually a drink) and as the meal empties from the stomach, acetaminophen is absorbed in the proximal small intestine and concentrations can then be measured in plasma (or saliva). The acetaminophen technique can only measure gastric emptying of liquids and is reliant on the assumption that the acetaminophen is rapidly absorbed in the duodenum (45). The use of the acetaminophen technique has provided misleading information relating to the effects of GLP-1RAs on gastric emptying, as shown by subsequent studies with scintigraphy; in some cases, the outcome of the acetaminophen test has been interpreted incorrectly (46-48). A recent review by Hiramoto et al (49). suggested that the magnitude of the delay in gastric emptying by GLP-1RAs is modest and had no effect to slow gastric emptying of liquids. However, this conclusion is based upon the use of the acetaminophen absorption technique and is, most likely, erroneous. For example, when measured using scintigraphy, the GLP-1RA lixisenatide was shown to markedly delay liquid emptying (50).

## Effect of Exogenous GLP-1, GLP-2, GIP, and Blockade of Endogenous GLP-1 on Gastric Emptying

Slowing of gastric emptying by infusions of exogenous native GLP-1, in pharmacological and modestly supraphysiological doses, has been observed in both healthy individuals and those with T2D (51-53), and the magnitude of slowing appears comparable in the 2 groups. In contrast, GIP has minimal, if any, effect on gastric emptying (54). GLP-1 receptors are present in the myenteric plexus (55) and are coupled to the G-protein G_Sα_ subunit. Agonist binding leads to adenylyl cyclase activation with consequent production of cAMP and inhibition of vagal activity (55). Consistent with the observation that exogenous GLP-1 slows gastric emptying, exendin 9-39 (a GLP-1 receptor antagonist) has been shown to increase antroduodenal contractility and reduce pyloric tone (56) to accelerate gastric emptying in health (57). This indicates that GLP-1 is a physiological modulator to slow the rate of gastric emptying, particularly as the acceleration of gastric emptying by exendin 9-39 occurs despite a concomitant increase in PYY secretion (58), which slows emptying (59). The slowing of gastric emptying induced by exogenous GLP-1 is greater when the baseline rate of gastric emptying is relatively more rapid (60) and in patients with critical illness, exogenous GLP-1 has been reported to not slow gastric emptying further when it is already delayed (60). In health, with sustained administration of GLP-1 (eg, continuous intravenous infusion for 24 hours), the slowing of gastric emptying by GLP-1, while still significant, is less marked (61). This provides evidence of tachyphylaxis to the slowing effect of GLP-1 even after only 4 to 24 hours of continuous exposure.

In addition to the postprandial effect to slow gastric emptying, exogenous GLP-1 in pharmacological doses may affect fasting motility to reduce phase III activity in the antroduodenojejunal region (62). This effect, observed in both animals (63) and humans (62), would be anticipated to increase the retention of larger nondigestible solids in the stomach. No studies are available regarding similar effects of GLP-1-based medications, nor is it known whether there may be tachyphylaxis to this effect.

Dipeptidyl-dipeptidase-4 (DPP-4) inhibitors block the breakdown of GLP-1 and GIP to improve insulin sensitivity and glycemic control. DPP-4 inhibitors, however, do not have a significant effect on gastric emptying (64) but also attenuate the postprandial PYY response by preventing the activation going from PYY_1-36_ (biologically inactive) to PYY_3-36_ (biologically active) (65). Because PYY_3-36_ slows gastric emptying, attenuation of the PYY response may counteract the effect of the increase in circulating GLP-1 to slow gastric emptying to explain why DPP-4 inhibitors have no effect on gastric emptying.

GLP-2 is co-secreted with GLP-1 following the exposure of the small intestine to nutrients. The primary function of GLP-2 is to regulate intestinal growth; however, there has predictably been interest in the effect of GLP-2 on gastric emptying. No effect of GLP-2 on gastric emptying was evident in studies using scintigraphy (66) and the stable isotope breath test (67). However, in an ultrasound study, GLP-2 was reported to slow gastric emptying modestly but to a much lesser extent than GLP-1 (68).

## Evidence for Slowed Gastric Emptying in Clinical Studies With GLP-1RAs and Tirzepatide

Numerous clinical studies have evaluated the effects of GLP-1RAs, as well as the dual GIP/GLP-1 RA tirzepatide, on gastric emptying as a primary or secondary endpoint in subjects with T2D and those with obesity but without diabetes (69). In such studies, particularly in T2D, a potential confounder is the concomitant reduction in blood glucose, which may affect gastric emptying independently (25). Exendin-4, the precursor to exenatide (the first GLP-1RA), lowered postprandial glucose levels in health (70).

**Figure 3.**
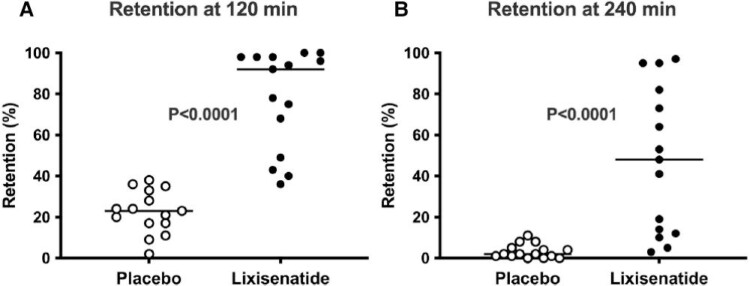
Intragastric retention of a 300-mL drink containing 75-g glucose at (A) 120 minutes and (B) 240 minutes after ingestion in 30 participants with T2D after 8 weeks treatment with either lixisenatide 20 µg (n = 15) or placebo (n = 15). Median values are indicated. Lixisenatide substantially slows gastric emptying of liquids at 120 minutes and 240 minutes. Reproduced from Kovoor et al (50). © 2024 The Authors. Published by Elsevier Inc.

Short-acting GLP-1RAs studied in people with T2D include exenatide BD (71, 72) and lixisenatide (73-76), with treatment durations spanning 5 days to 14 weeks. In these studies, gastric half-emptying times of liquids and/or solids were shown to be variably increased up to 3.4-fold, compared to placebo (71). Furthermore, marked slowing of gastric emptying may occur in doses substantially less than those used for glucose lowering in T2D (71) to slow emptying of both nutrient liquids and solids (50). For example, in 15 individuals with T2D, the intragastric retention of a 75-g oral glucose drink (measured using scintigraphy) was much greater with individuals treated with 20 µg lixisenatide when compared to placebo at both 120 and 240 minutes (50) (Fig. 3). As is the case with native GLP-1, the slowing of gastric emptying induced by both exenatide BD and lixisenatide is greater when the baseline rate of gastric emptying is relatively faster and is predictive of the reduction in postprandial glucose (71, 77).

In the majority of studies (Table 1), there was a reduction in energy intake with both short- and long-acting GLP-1RAs. However, there does not appear to be a relationship between the magnitude of the decrease in energy intake and the delay in gastric emptying (35, 82).

**Table 1.** Effect of GLP-1RAs on energy intake and its relationship to gastric emptying

Target population/study (first author, year)	Compound/preparation	Final dose(s)	Duration	Comparison	Outcome of GLP-1RA treatment on energy intake (⇓ decreased, ⇔ no change)	Comments
**Type 2 diabetes**						
Bae et al (78)	Lixisenatide (n = 29)	10 µg SC daily	Acute	Saline (n = 29)	⇓	
Flint et al (79)	Liraglutide (n = 18)	1.8 mg SC daily	3 weeks	Placebo (n = 18) (details not specified)	⇓	
Gibbons et al (80)	Semaglutide (n = 13)	14 mg Oral daily	12 weeks	Placebo (n = 13)	⇓	
Horowitz et al (81)	Liraglutide (n = 46)	1.8 mg SC daily	4 weeks	Placebo (n = 46)	⇔	Acetaminophen concentration AUC_0-60 minutes_ and C(max) were less with liraglutide vs placebo
Jalleh et al (35)	Lixisenatide (Health n = 15) (T2D n = 15)	10 µg SC daily	Acute	Placebo (n = 30)	⇓	Lixisenatide delayed gastric emptying (scintigraphy) but the reduction in energy intake by lixisenatide was unrelated to changes in gastric emptying or intragastric distribution
Quast et al (82)	Lixisenatide (n = 24) Liraglutide (n = 26)	20 µg SC daily 1.8 mg SC daily	10 weeks	Lixisenatide vs liraglutide	⇓	Both lixisenatide and liraglutide reduced energy intake comparably. Weight loss and appetite reduction were not related to the delay in gastric emptying (isotope breath test) or gastrointestinal adverse effects
Valenzuela-Vellejo et al (83)	Liraglutide (n = 20)	1.8 mg SC daily	17 days	Placebo (n = 20)	⇔	
**Obesity**						
Acosta et al (84)	Exenatide (n = 10)	5 µg SC BID	30 days	Placebo (n = 10)	⇔	
Basolo et al (85)	Exenatide (n = 40)	10 µg SC BID	24 weeks	Placebo (n = 39)	⇓	
Blundell et al (86)	Semaglutide (n = 30)	1 mg SC weekly	12 weeks	Placebo (n = 30)	⇓	
Gabe et al (87)	Semaglutide (n = 30)	50 mg oral daily	20 weeks	Placebo (n = 31)	⇓	No difference in time to Cmax, AUC_0-5h_, or AUC_0-1h_ for paracetamol concentrations. Relationship between acetaminophen concentration and energy intake was not analyzed
Friedrichsen et al (88)	Semaglutide (n = 36)	2.4 mg SC weekly	20 weeks	Placebo (n = 36)	⇓	No difference in time to maximum acetaminophen concentration. Relationship between acetaminophen concentration and energy intake was not analyzed
Schlögl et al (89)	Exenatide (n = 24)	0.12 pmol/kg/min IV	Acute	Placebo (n = 24)	⇓	
Shoemaker et al (90)	Exenatide (n = 23)	2 mg SC weekly	36 weeks	Placebo (n = 19)	⇓	Hypothalamic obesity with suprasellar tumors
Silver et al (91)	Liraglutide (n = 44)	1.8 mg SC daily	14 weeks	Sitagliptin (n = 22) Caloric restriction (n = 22)	⇔	Obesity with prediabetes
van Can et al (92)	Liraglutide (n = 30)	1.8 mg SC daily	5 weeks	Placebo (n = 30)	⇓	
**Type 1 diabetes**						
Dubé et al (93)	Liraglutide (n = 15)	1.8 mg SC daily	24 weeks	Placebo (n = 15)	⇓	

Abbreviations: BID, twice daily; SC, subcutaneous; T2D, type 2 diabetes.

### Potential Impact of Slower Gastric Emptying Induced by GLP-1RAs—Risk of Hypoglycemia

In individuals with insulin-treated diabetes, the postprandial insulin dose is often based on the amount of carbohydrate consumed, the “insulin: carbohydrate ratio.” A limitation of this approach is that it does not take into account the gastrointestinal contribution to postprandial glycemia, particularly gastric emptying. Because gastric emptying is not routinely measured, information about its rate in a specific individual is not usually available to the treating clinician. This is despite compelling evidence that modulating gastric emptying may have a major impact on postprandial glycemia. For example, in T2D, when gastric emptying is slowed using a drug (such as morphine), or by dietary modifications such as a “preload” (a small nutrient load such as olive oil or whey protein taken ∼15 minutes before the meal), the glycemic excursion is substantially less (42, 94, 95). Abnormally delayed gastric emptying, sometimes associated with clinical symptoms (ie, “diabetic gastroparesis”), occurs in ∼30% of people with longstanding, suboptimally controlled, insulin-treated diabetes. Gastrointestinal autonomic neuropathy is, at least in part, responsible for the impairment in gastric emptying, which increases the propensity for a mismatch between onset of action of subcutaneously administered prandial insulin and the absorption of ingested carbohydrates from the small intestine. In an early study, Ishii et al (96) found that the insulin requirement to maintain euglycemia postprandially was less in insulin-treated people with gastroparesis than those without gastroparesis during the first 120 minutes following a mixed meal containing 60 g carbohydrate but was greater between 180 and 240 minutes. The implication is that delayed gastric emptying reduces the initial insulin requirement following a meal (96), and when markedly delayed, it may increase the propensity for insulin-induced hypoglycemia. This concept is supported by a study by Lysy et al (97), who reported that gastric emptying (measured using a stable isotope breath test) was frequently delayed in people with insulin-treated diabetes and “unexplained” (usually postprandial) hypoglycemia. We have proposed the term “gastric hypoglycemia” to describe this phenomenon (98).

### Gastric “Counterregulation” of Hypoglycemia—Impact of GLP-1RAs

Acute hypoglycemia is the most feared adverse event in people treated with insulin or sulfonylureas (99). The stomach plays a key, but often overlooked, role in the counterregulation of insulin-induced hypoglycemia because of a marked acceleration of gastric emptying, which increases the rate of carbohydrate absorption (100). The earliest evidence to support its importance was reported as early as 1924 in dogs by Bachrach (100), barely 3 years after the discovery of insulin.

*The activity of the stomach during hypoglycemia suggests an adaptation reaction in that it prepares the organism for rapid digestion of food, the ingestion of which is imperative to restore the lowered blood sugar*. Bachrach, 1953 (101)

Insulin-induced hypoglycemia accelerates gastric emptying substantially, even in individuals with gastroparesis and autonomic neuropathy (102, 103). Antecedent hypoglycemia (blood glucose 2.8 mmol/L) does not appear to attenuate the acceleration of gastric emptying by subsequent insulin-induced hypoglycemia in health (104). The magnitude of acceleration of gastric emptying is dependent on the degree of hypoglycemia (ie, in health), the acceleration of gastric emptying is greater when the blood glucose is clamped at 2.6 mmol/L than 3.6 mmol/L (103).

The risk of hypoglycemia with GLP-1RA monotherapy is predictably very low or even nonexistent because the insulinotropic actions are highly glucose-dependent (105-107). In contrast, clinical trials and real-world studies are indicative of a slightly increased risk of hypoglycemia when GLP-1RAs are combined with insulin or sulfonylureas (108, 109). Accordingly, it is of interest that the acceleration of gastric emptying induced by hypoglycemia is attenuated by administration of native GLP-1 (110) and the short-acting GLP-1RA, exenatide (111), which may well increase the potential for a mismatch between nutrient delivery after meal ingestion and insulin action in people co-prescribed a GLP-1RA and insulin, thus predisposing to hypoglycemia. This issue warrants further evaluation. Furthermore, novel incretin-based therapies under evaluation stimulate not only receptors for GLP-1 but also glucagon (4) and amylin (112), both known to delay gastric emptying.

### Duration of Slowing of Gastric Emptying by GLP-1RAs

In studies evaluating the effects of long-acting GLP-1RAs on gastric emptying in T2D, liraglutide (72-74, 81, 113-115), efpeglenatide (115), and oral semaglutide (47) have been examined over treatment durations of up to 3 months (115). In these studies, gastric emptying appears to be less markedly slowed than with short-acting GLP-1RAs, with a maximum increase in the mean gastric half-emptying time of ∼1.6-fold (116). However, the methodology used to quantify gastric emptying (eg, acetaminophen absorption) in many cases was suboptimal. In line with these findings, the dual GIP/GLP-1 RA, tirzepatide, was found to delay gastric emptying in T2D, with evidence that this effect may be diminished after 23 days of treatment (ie, suggestive of tachyphylaxis) (Table 2) (117).

**Table 2.** Effects of GLP-1RAs and combination medication on gastric, small intestinal, and gallbladder motility

GLP-1 based medication	Effect on gastric emptying	Effect on small intestinal motility	Effect on gallbladder emptying
**Short-acting GLP-1RA**			
Exenatide BID (84, 118, 119)	⇓⇓	⇓	⇓
Lixisenatide (77, 120)	⇓⇓	⇓ (Case reports)	⇓
**Long-acting GLP-1RA**			
Liraglutide (73, 121, 122)	⇓	⇓	⇓
Dulaglutide (123, 124)	⇓	⇓ (Case reports)	Not studied
Albiglutide (125, 126)	Not studied	⇓ (Case reports)	⇓
Semaglutide SC (46, 126)	⇓	⇓ (Case reports)	Not studied
Semaglutide PO (47, 87)	⇓/⇔	Not studied	Not studied
**Combined GLP-1-based medication**			
Tirzepatide (GLP-1/GIP co-agonist) (117, 127)	⇓	⇓ (Case reports)	Not studied
Retatrutide (GLP-1/GIP/glucagon co-agonist) (117)	⇓ (Animal study)	Not studied	Not studied

In individuals with obesity but not T2D, only long-acting GLP-1RAs have been evaluated. These studies used liraglutide (128, 129), once-weekly exenatide (130), and oral/subcutaneous semaglutide (46, 87, 88, 131) with treatment durations of up to 20 weeks (88). Although a delay in gastric emptying has been reported in studies of shorter treatment durations (8-16 weeks), there was no effect on gastric emptying with high-dose semaglutide (2.4 mg per week) or oral semaglutide (50 mg daily) in 2 studies evaluating the longest treatment duration (20 weeks). These observations should, however, be viewed circumspectly because the acetaminophen technique was used (88). A study by Maselli et al using scintigraphy indicated a persisting marked delay in gastric emptying after 16 weeks of liraglutide therapy (3 mg per day), albeit less than the delay at 5 weeks (129). However, this study also confirmed the expected substantial interindividual differences in the magnitude of the delay in gastric emptying induced by GLP-1RAs (116). As with studies in T2D, the slowing of gastric emptying by liraglutide was greater when the rate of gastric emptying at baseline was more rapid and was predictive of weight loss (129).

In summary, short- and long-acting GLP-1RAs slow gastric emptying in individuals with T2D, or obesity without T2D, an effect integral to postprandial glucose lowering and, potentially, weight loss. Slowing of gastric emptying is variable, depends on the baseline rate of gastric emptying, and with long-acting GLP-1RAs, may diminish with sustained administration. Prospective studies of the effects of long-acting GLP-1RAs on gastric emptying using sensitive methodology are required to clarify this important issue.

## Effects of GLP-1RAs on the Gallbladder and Biliary Tract

Since the introduction of GLP-1RAs, there have been reports of gallbladder disorders associated with their use (132, 133). Whether this represents a class-effect is difficult to determine, given that both T2D and rapid weight loss independently increase the risk of gallstone-related pathology (134, 135). In a meta-analysis of 10 prospective studies including more than 220 000 cases, the relative risk for individuals with diabetes (T1D or T2D) having gallstones, a cholecystectomy, or cholecystitis was 1.56 (95% CI, 1.26-1.93) compared to individuals without diabetes (134). Prescribing information for GLP-1RAs was amended in 2022 to include warnings regarding the potential for gallbladder and biliary disease (136), following the publication of a systematic review and meta-analysis by He et al of 76 randomized controlled trials comparing the use of GLP-1RAs with placebo or a non-GLP-1RA glucose-lowering drug in more than 100 000 individuals (137). Randomization to GLP-1RA treatment was found to be associated with increased risks of cholelithiasis (relative risk [RR], 1.27; 95% CI, 1.10-1.47), cholecystitis (RR, 1.36; 95% CI, 1.14-1.62) and biliary disease (RR, 1.55; 95% CI, 1.08-2.22) (137). An increased risk of GLP-1RA-associated gallbladder disorders was only evident with higher doses and longer durations of treatment (>26 weeks), and this risk was greatest when GLP-1RAs were prescribed for weight loss (137). However, an increased risk of gallbladder or biliary disease was not evident with all GLP-1RAs: liraglutide and dulaglutide were associated with an increased risk and point estimates favored an increased risk with exenatide and subcutaneous semaglutide that was not statistically significant. There was, however, no evidence of increased risk with either lixisenatide, albiglutide, or oral semaglutide. Previously, only 12 trials have reported biliary tract cancer as an outcome and in all cases, the durations of follow-up were short. The overall incidence was, however, low (<1/1000) and there was no suggestion of an increased risk associated with GLP-RA use (137).

**Figure 4.**
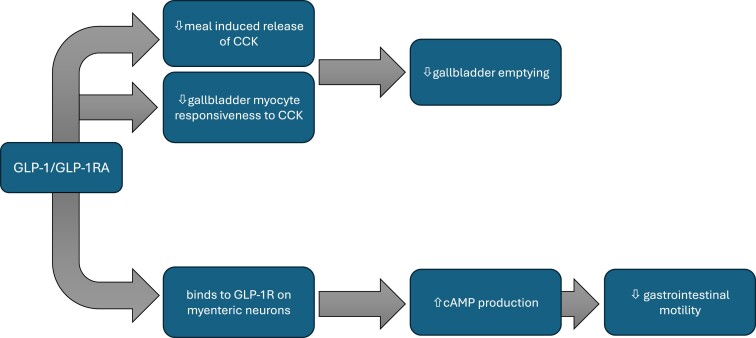
Mechanisms for the effect of GLP-1/GLP-1RA on gallbladder and gastrointestinal motility.

Several small trials have provided insights into the effects of supraphysiological/pharmacological concentrations of GLP-1 on gallbladder motor function which provide a mechanistic rationale for GLP-1RA-associated harm. For example, in healthy volunteers, Keller et al reported that a single subcutaneous dose of short-acting exenatide (10 µg) reduced CCK-induced gallbladder contractility in the fasted state by ∼30% (118). Utilizing similar study designs in healthy volunteers, gallbladder emptying induced by IV CCK was shown to be attenuated by single subcutaneous doses of albiglutide 50 mg (125) and lixisenatide 20 µg (120). In a crossover study of 10 healthy volunteers and 10 participants with T1D, Rehfeld et al reported that an intravenous infusion of GLP-1, at a pharmacological dose, suppressed meal-induced release of CCK and attenuated meal-induced gallbladder contractility substantially (138). Accordingly, these short-term studies in healthy individuals indicate that GLP-1-based therapy has the potential to attenuate both the CCK response to meals and the responsiveness of the gallbladder to CCK, although the underlying mechanism of this remains unknown (Fig. 4). Limitations include a lack of studies in obese or T2D patients or with prolonged use of a GLP-1RA. Nevertheless, given that impaired gallbladder contraction is known to predispose to sludge and gallstone formation, it is reasonable to suggest that long-term use of GLP-1RAs could predispose to gallbladder and biliary disease (138).

A signal for harm has not been evident in trials of long-term GLP-1RA use in target populations. For example, in a randomized placebo-controlled trial in 60 patients with T2D, Smits et al investigated the effects of 12 weeks of subcutaneous liraglutide (1.8 mg/day) and concluded it had no effect on either fasting gallbladder volume or gallbladder emptying (139), whereas in a randomized placebo-controlled trial in 52 obese patients, a higher daily dose of liraglutide (3 mg) for 8 weeks had no effect on maximum gallbladder contraction, but delayed the time to full contraction and delayed refilling (121). The inconsistency in the observed biliary effects of liraglutide in these studies may, in part, reflect the different doses used.

However, there are also potential beneficial effects of the inhibition of gallbladder emptying. It may reduce the availability of bile in the small intestine for emulsification and digestion of dietary lipids and, therefore, reduce the postprandial rise in lipids as detailed in the following section.

In summary, observational data from large randomized controlled trials is indicative of a modestly increased incidence of gallbladder disease with GLP-1RA use. Although studies quantifying the acute effects of GLP-1RAs on gallbladder motility have demonstrated attenuation of contractility by GLP-1RA, this has not been clearly shown with longer term use in target populations, particular at lower doses.

### GLP-1RAs and Postprandial Triglycerides and ApoB-48

In rats, GLP-1 inhibits lymph flow and triglyceride and ApoB-48 concentrations in lymphatic fluid following an intestinal lipid load (140), supporting a direct effect of GLP-1 signaling to regulate intestinal chylomicron formation. Consistent with this concept, even DPP-4 inhibitors, which increase active GLP-1 concentrations modestly but have no effect on gastrointestinal motility, may reduce postprandial triglyceride and ApoB48 levels substantially (141, 142). The reduction in the postprandial rises in triglycerides and plasma nonesterified fatty acid levels by IV GLP-1 may also relate to slowing of gastric emptying (143).

All GLP-1RAs (144), as well as the GLP-1/GIP dual RA, tirzepatide (145), reduce postprandial triglyceride and ApoB-48 levels significantly, which may be relevant to their cardiovascular benefits. Slowing of gastric emptying and small intestinal transit may reduce the delivery of dietary lipids for subsequent synthesis of chylomicrons in enterocytes.

## Effects of GLP-1 and GLP-1RAs on Intestinal Motility

Constipation and diarrhea are well-recognized adverse effects of GLP-1RAs (146). The physiological effect of GLP-1 on small intestinal motility is inhibitory, consistent with a contribution to the “ileal brake,” the phenomenon by which the presence of nutrients in the distal small intestine induces negative feedback on the stomach and proximal small intestine (147) to slow gastrointestinal transit. In healthy humans, IV infusion of the GLP-1RA, exendin 9-39, has been reported to increase duodenal motility during small intestinal nutrient infusion (148). In contrast, IV infusions of native GLP-1, resulting in physiological or supraphysiological plasma GLP-1 concentrations, inhibits phase III of the migrating motor complex in the antroduodenaljejunal region (62) and suppresses duodenal pressure waves both during fasting and following intraduodenal lipid infusion (149). Similarly, acute administration of GLP-1RAs (subcutaneous liraglutide and IV exenatide) inhibits small intestinal motility and slows small intestinal transit in both healthy volunteers and those with T2D (119, 122). This inhibition of small intestinal motility induced by GLP-1 and GLP-1RAs may be mediated by vagal afferents, as well as a direct action on the central nervous system (150-152).

When compared to the small intestine, even less is known about the effects of GLP-1 and GLP-1RAs on colorectal function. A patient with a neuroendocrine tumor secreting large amounts of GLP-1 and GLP-2 exhibited delayed transit through the gastrointestinal tract, including the colon (153), whereas the use of GLP-1RAs has recently been reported to be associated with lower quality bowel preparation for colonoscopy (154). In contrast, acute administration of the GLP-1RA, ROSE-010, in women with constipation-predominant irritable bowel syndrome showed that colonic transit was accelerated, rather than delayed (155).

A number of case reports have linked the use of GLP-1RAs (123, 156), and the dual GIP-/GLP-1 receptor agonist tirzepatide (127), to bowel obstruction or ileus. Several studies (126, 157-161) utilizing large medical databases have subsequently systematically investigated whether such an association exists and the majority of studies suggest that there is an association. However, in these studies, use of DPP-4 inhibitors was, in general, associated with a higher risk of intestinal obstruction than GLP-1RAs (126, 157, 161, 162), which is surprising given the minimal effect of GIP on gastric emptying and the marginally elevated GLP-1 concentrations observed (54, 163).

It is clear that more information is needed to define the effects of GLP-1RAs on both small and large intestinal motility (146), including whether these are susceptible to tachyphylaxis with repeated dosing, as may be the case with slowing of gastric emptying.

## Conclusion

GLP-1RA and GLP-1/GIP co-agonist therapies may have major effects on gastrointestinal and biliary tract motility. An improved understanding is likely to facilitate more personalized use and will inform the development of evidence-based clinical guidelines.

## Data Availability

Data availability is not applicable to this article as no new data were created in this review.

## Abbreviations

3D: 3-dimensional
CCK: cholecystokinin
DPP-4: dipeptidyl-dipeptidase-4
GLP-1: glucagon-like peptide-1
PYY: peptide YY
RA: receptor agonist
T1D: type 1 diabetes
T2D: type 2 diabetes
